# Heterotypic Cellular Interactions in the Ovarian Tumor Microenvironment: Biological Significance and Therapeutic Implications

**DOI:** 10.3389/fonc.2014.00018

**Published:** 2014-02-06

**Authors:** Honami Naora

**Affiliations:** ^1^Department of Molecular and Cellular Oncology, University of Texas MD Anderson Cancer Center, Houston, TX, USA

**Keywords:** ovarian cancer, tumor microenvironment, mesothelium, endothelial cells, adipocytes, fibroblasts, mesenchymal stem cells, macrophages

## Abstract

The majority of women who are diagnosed with epithelial ovarian cancer present with extensive peritoneal carcinomatosis and are rarely cured by conventional chemotherapy. Ovarian cancer cells typically disseminate by shedding into the peritoneal fluid and implant on the mesothelium-lined peritoneal surfaces that overlie connective and white adipose tissues. Emerging evidence indicates that ovarian tumor progression is orchestrated by dynamic interplay between tumor cells and a variety of stromal cells such as adipocytes, endothelial cells, fibroblasts, mesenchymal stem cells, macrophages, and other immune cells. This mini-review discusses the biological significance of the heterotypic cellular interactions in the ovarian tumor microenvironment and the therapeutic implications of targeting these interactions.

## Introduction

The lethality of epithelial ovarian cancer primarily stems from late diagnosis. Women who are diagnosed with early-stage, ovarian-confined tumors have a 5-year survival rate of more than 90% (1). However, 60% of ovarian cancer patients present with advanced-stage, disseminated disease, and these women have a 5-year survival rate of less than 30% (1). Despite optimal tumor-debulking surgery and initial high response rates to platinum–taxane chemotherapy (70–80%), most patients with advanced-stage ovarian cancer relapse within 18 months (2). The biological behavior of ovarian cancer differs markedly from the hematogenous or lymphatic metastasis found for many other types of tumors. Ovarian cancer can initially progress by extending to adjacent pelvic tissues, but mainly disseminates by shedding into the peritoneal fluid, which transports tumor cells throughout the peritoneal cavity (3–5). These cells then implant on the surfaces of the cavity wall and abdominal organs. The omentum, a fat pad that extends from the stomach and suspends over the bowel, is the most frequently involved site (3–5). Seeding of the peritoneal cavity with tumor cells is often associated with ascites. It is increasingly recognized that progression of virtually all types of tumors is dynamically controlled by cross-talk between tumor cells and stromal cells (6, 7). As discussed below, the peritoneal cavity is a conducive environment for carcinomatosis, and the receptors and ligands that mediate interactions between ovarian cancer cells and stromal cells are candidate targets for new-generation therapies. This article is not intended as an exhaustive review of therapies, but provides an overview of the major cellular constituents of the ovarian tumor microenvironment, the complexity of their regulation, and focal points for therapeutic intervention.

## Mesothelial Cells

**Figure 1:**
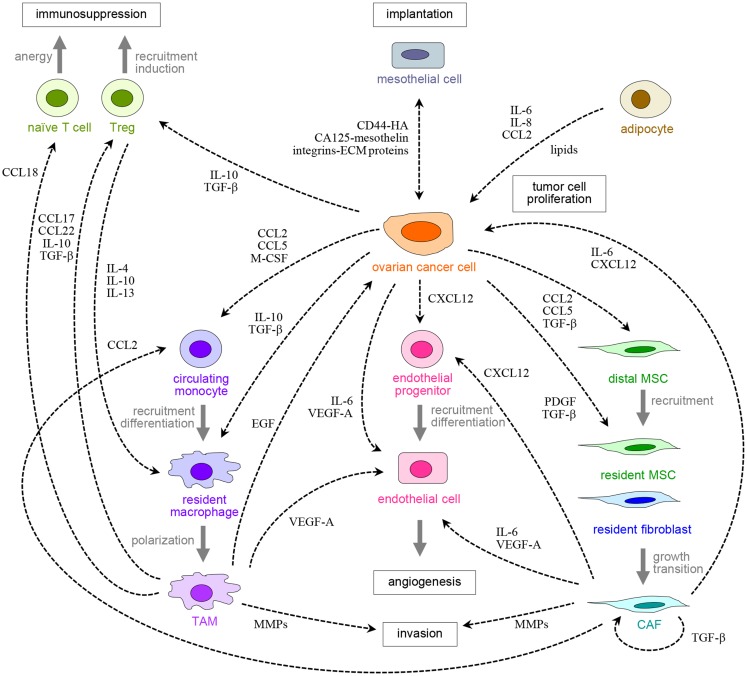
**Peritoneal carcinomatosis is orchestrated by cross-talk between ovarian cancer cells, resident peritoneal cells, and other host cells that are recruited to tumors**. Examples of receptors and ligands that facilitate these reciprocal cellular interactions are shown.

Mesothelial cells are of mesodermal origin and form a protective monolayer that lines peritoneal, pleural, and pericardial surfaces (8). Interactions between ovarian cancer cells and peritoneal mesothelial cells are mediated by a variety of cell surface molecules (Figure 1). The ovarian cancer biomarker CA125 has been implicated in facilitating tumor cell implantation by its ability to bind mesothelin that is expressed by mesothelial cells (9). Gonadotropin-releasing hormone receptor signaling stimulates ovarian cancer cell attachment to mesothelial cells in part by inducing P-cadherin that is expressed in ovarian cancer cells and in mesothelial cells (10). Several integrins mediate attachment of ovarian cancer cells to mesothelial cells and/or facilitate tumor cell interactions with the submesothelial extracellular matrix (ECM) (11–15). Iwanicki and colleagues identified that spheroids of ovarian cancer cells displace mesothelial cells to gain access to the underlying stroma by using myosin-generated mechanical force that is dependent on α5β1 integrin and talin I (16). Mesothelial breach has also been found to be facilitated by CD157, a glycoprotein that is expressed in normal mesothelium and in 93% (82/88 cases) of ovarian cancers (17).

Because the mesothelium is the first point-of-contact for floating ovarian cancer cells at distal sites, targeting molecules that promote tumor–mesothelial interactions is a potential strategy to impede disease progression. Studies of the glycoprotein CD44 highlight several limitations of this approach. CD44 is expressed in ovarian cancers and binds hyaluronic acid (HA), a glycosaminoglycan that is synthesized by mesothelial cells (18). Strobel and colleagues found that treatment with neutralizing monoclonal antibody (mAb) to CD44 inhibited the number of peritoneal implants by 70% in ovarian cancer xenograft models, but did not reduce growth rates of tumors (19). Blocking tumor cell implantation alone might therefore not be therapeutically efficacious. Furthermore, neutralization of CD44 did not completely block implantation (19). Other studies have also shown that interactions between ovarian cancer cells and mesothelial cells are only partially inhibited by mAbs to a single adhesion molecule (13–16). In a study by Cannistra and colleagues, CD44 was detected in 94% (15/16 cases) of solid ovarian tumor tissues but in only 25% (2/8 cases) of ascitic tumor cells (18). To effectively block tumor cell implantation, it is likely that multiple adhesion molecules need to be targeted and these molecules need to be highly expressed on free-floating tumor cells.

## Endothelial Cells

Tumor growth depends on the development of a neovasculature that supplies oxygen, nutrients, and growth factors. Increased angiogenesis as manifested by high tumor microvessel density has been found by several studies to be predictive of poor outcomes in ovarian cancer patients (20–22). Angiogenesis is a dynamic process orchestrated by pro- and anti-angiogenic factors that control recruitment of endothelial progenitors, growth and maturation of endothelial cells, and organization of endothelial cells into tubular structures (23, 24). Ovarian cancers express a variety of pro-angiogenic factors including the vascular endothelial growth factors (VEGF), fibroblast growth factor (FGF)-2, interleukin (IL)-6, IL-8, angiopoietin, and platelet-derived growth factor (PDGF) (25). Stromal fibroblasts and macrophages are also rich sources of pro-angiogenic factors (Figure 1). VEGF-A has emerged as the predominant pro-angiogenic factor in ovarian cancer (25) and is also the causative factor of ascites formation (26).

Agents that target VEGF signaling have been the focus of intensive clinical investigation in ovarian cancer. One major class of agents includes ligand inhibitors. Aflibercept is a fusion protein that combines the Fc portion of human IgG1 with the principal ligand-binding domains of VEGF receptor (VEGFR)-1 and VEGFR-2 (27). Aflibercept is generally well-tolerated, but the endpoint of a >5% response rate was not reached in a Phase II study of aflibercept in patients with recurrent ovarian cancer (28). Bevacizumab is a humanized mAb that neutralizes all forms of VEGF. Two phase II studies (GOG 170D and AVF 2949g) evaluated bevacizumab as a single agent in patients with recurrent ovarian cancer and reported response rates of 21.0 and 15.9%, respectively (29, 30). Combining bevacizumab with carboplatin and paclitaxel increased progression-free survival (PFS) by ~3.6 months as compared to standard chemotherapy alone in two phase III studies of patients with recurrent ovarian cancer (31, 32). Tyrosine kinase inhibitors (TKIs) are another class of agents that has attracted substantial interest. Sorafenib inhibits several receptor tyrosine kinases including VEGFR-2, VEGFR-3, PDGF receptor (PDGFR)-β, c-kit and Flt-3, and also RAF serine/threonine kinases (33). In a phase II trial of sorafenib, only 2 of 59 evaluable ovarian cancer patients had partial responses (34). Several TKIs that inhibit all three VEGFRs, both PDGFRs and also the FGF receptor have been undergoing clinical trials in ovarian cancer patients and are discussed in several recent articles (35–37).

## Adipocytes

Omental, mesenteric, and gonadal tissues are major repositories of visceral white adipose tissues and are frequently colonized by ovarian cancer cells (3, 4). Adipocytes (fat cells) are the predominant component of adipose tissue. Adipocytes promote proliferation of breast, colon, and prostate cancer cells and this stimulatory effect is mediated in part by the adipokine leptin (38–40). Leptin also stimulates ovarian cancer cell growth (41). The mechanism by which adipocytes promote ovarian cancer growth is a relatively new area of investigation. Nieman and colleagues identified that omental adipocytes secrete IL-6, IL-8, chemokine (C-C motif) ligand 2 (CCL2), and tissue inhibitor of metalloproteinases-1, and that mAbs to each of these factors inhibited chemotaxis of ovarian cancer cells toward adipocytes by at least 50% (42). Using co-cultures of omental adipocytes and ovarian cancer cells, the authors found that adipocytes stimulate tumor cell proliferation by directly transferring lipids to tumor cells (42). They also identified that fatty acid-binding protein 4 (FABP4), a lipid transporter, is more highly expressed in omental metastases than in primary ovarian tumors (42). Furthermore, the number of metastatic nodules that developed in a *Fabp4-*deficient orthotopic model of ovarian cancer was only 2% of the number of metastatic nodules that developed in the wild-type model (42). This elegant study demonstrated that adipocytes recruit ovarian cancer cells and support tumor growth through provision of energy (Figure 1), and raises the possibility that targeting lipid metabolism and/or trafficking could be a strategy to impede peritoneal growth and spread of ovarian cancer.

## Cancer-Associated Fibroblasts

Cancer-associated fibroblasts (CAFs) are a predominant component of the tumor stroma and have a profoundly negative impact on outcomes of cancer patients (7, 43). CAFs are often distinguished from normal fibroblasts by their expression of markers of myofibroblasts and activated fibroblasts such as α-smooth muscle actin (αSMA) and fibroblast activation protein (FAP) (7, 43). CAFs derive from various cell types. Endothelial-to-mesenchymal transition has been identified as a source of CAFs in mouse models of melanoma and pancreatic cancer (44). CAFs can also derive from breast cancer cells that have undergone epithelial-to-mesenchymal transition (45). A study in which xenografts were generated from green fluorescent protein (GFP)-transfected ovarian cancer cells found that virtually all αSMA+ stromal cells lacked GFP, suggesting that CAFs did not derive from ovarian cancer cells (46). Tissue-resident fibroblasts are a major source of CAFs (43, 47) (Figure 1). Ko and colleagues demonstrated that ovarian cancer cells induce normal omental fibroblasts to express CAF markers and mitogenic factors such as IL-6 and chemokine (C-X-C motif) ligand 12 (CXCL12) that stimulated tumor cell proliferation (46). Overexpression of the patterning gene *HOXA9* increased the CAF-promoting ability of ovarian cancer cells by activating the expression of transforming growth factor-β2 (TGF-β2). In turn, TGF-β2 acted in a paracrine manner on omental fibroblasts and stimulated a TGF-β auto-regulatory loop in the stroma (46). Inhibition of ovarian cancer cell-derived TGF-β2 in xenograft models reduced the number of αSMA+ stromal cells in omental implants by 90% and the tumor mitotic activity by 75% (46). These findings support a model in which ovarian cancer cells “educate” omental fibroblasts to become permissive for tumor growth. Studies of Mitra and colleagues indicate that this programing is controlled in part by specific microRNAs. These authors identified differences in microRNA expression patterns in normal omental fibroblasts and in CAFs isolated from omental tumors, and demonstrated that altering expression of three microRNAs (miR-31, miR-155, miR-214) induces normal fibroblasts into CAFs (48).

Mesenchymal stem cells (MSCs) are adult stem cells that can differentiate into the osteogenic, myogenic, chondrogenic, and adipogenic lineages, and are another source of CAFs. Studies using animal models of ovarian cancer and other solid tumors have shown that bone marrow-derived MSCs home to tumors and transition into CAFs (49–51). White adipose tissues contain abundant MSCs that have multi-potency comparable to that of bone marrow MSCs (52). Ovarian cancer cells induce normal adipose MSCs to acquire features of CAFs (46). Lysophosphatidic acid is abundant in ovarian cancer ascites and induces CAF features in adipose MSCs by stimulating TGF-β signaling (53). Because of the propensity of ovarian cancer to involve adipose tissue-rich peritoneal sites, adipose MSCs could be a significant source of CAFs in this disease. Normal cells that express CAF markers have been detected in omental tissues of ovarian cancer patients without overt omental metastasis (54). This raises the intriguing possibility that tumor-derived factors fertilize the omental “soil” before tumor cells implant.

Cancer-associated fibroblasts express many pro-angiogenic growth factors, ECM molecules, and matrix metallo-proteinases (MMPs) (7, 43). CAFs stimulate ovarian cancer cell invasiveness and the abundance of CAFs in ovarian cancers correlates with microvessel density (54). Omental fibroblasts that are stimulated by ovarian cancer cells have been found to secrete levels of VEGF-A and IL-6 that are, respectively, 5- and 10-fold higher than the levels secreted by unstimulated fibroblasts (46). A study by McLean and colleagues revealed that CAFs might drive ovarian tumor progression by expanding the cancer stem cell pool. These authors identified that propagating ovarian cancer cells with MSCs isolated from ovarian tumor tissues increased the number of cancer stem cells and that this enhancement was due in part to MSC-derived bone morphogenetic protein 2 (55).

Because CAFs express growth factors that stimulate tumor cell proliferation, metastasis, and angiogenesis (Figure 1), one strategy to inhibit the tumor-promoting ability of CAFs is to use agents that neutralize these growth factors. Another approach is to prevent normal fibroblasts and MSCs from transitioning into CAFs by inhibiting TGF-β signaling. A number of TGF-β inhibitors, such as ligand traps, antisense oligonucleotides, and TGF-β type I receptor (TGFβRI) kinase inhibitors, have been evaluated in pre-clinical and clinical studies (56, 57). Cai and colleagues found that treating mice with the TGFβRI inhibitor A83-01 reduced the abundance of αSMA+ stromal cells in ovarian tumor xenografts by 50% but did not increase survival (58). CAFs express PDGFRs (43) and could be inhibited by TKIs that target these receptors. Several studies have targeted the serine protease FAP. Depletion of FAP inhibited stromagenesis, tumor growth, and angiogenesis in mouse models of lung and colon cancers (59). A FAP mAb has been found to be well-tolerated but failed to show efficacy in a clinical trial of patients with colorectal cancer (60). A prodrug that consists of a FAP-specific peptide coupled to a cytotoxic analog of thapsigargin, induced stromal cell death in prostate and breast tumor xenografts and decreased tumor volumes by ~70% (61).

## Tumor-Associated Macrophages and Other Immune Cells

Tumor-associated macrophages (TAMs) are the major immune component of the tumor stroma and derive from monocyte precursors that are recruited to tumors (6, 62–64). Ovarian cancer cells express factors that stimulate monocyte chemotaxis and maturation such as CCL2 and macrophage colony stimulating factor (M-CSF) (65, 66). Analogous to the Th1/Th2 dichotomy of T cell responses, macrophages exhibit polarized phenotypes in response to different signals. Stimulation of macrophages with microbial agents or interferon-γ induces an M1 phenotype that is characterized by expression of immunostimulatory cytokines. In contrast, stimulation with IL-4, IL-10, or IL-13 induces an M2 phenotype that is characterized by the expression of immunosuppressive cytokines (62, 63). It is widely recognized that TAMs exhibit an M2 phenotype and that normal macrophages are “educated” by tumor cells to transition into TAMs (62–64) (Figure 1). Macrophages polarize toward an M2 phenotype when stimulated with ovarian cancer ascites (67, 68). This polarization was initially attributed to IL-10 because ascites contain only low levels of IL-4 and IL-13 (62). However, IL-6 and leukemia inhibitory factor (LIF) are present at high levels in patient ascites and also induce differentiation of monocytes into TAMs (67). It has also been recently shown that ovarian tumor-derived TGF-β2 and CCL2 stimulate normal peritoneal macrophages to acquire features of TAMs (69).

TAMs are strongly associated with poor outcomes in cancer patients (64). Studies of breast cancer have revealed that TAMs are rich sources of epidermal growth factor (EGF), MMPs, and pro-angiogenic factors such as VEGF-A (70, 71). An important mechanism by which TAMs promote tumor progression is by suppressing adaptive immunity. TAMs have poor antigen presentation capability and highly express IL-10, TGF-β, CCL17, CCL18, and CCL22 (62, 63). IL-10 and TGF-β inhibit dendritic cell maturation and T cell proliferation (62, 63). CCL18 induces naïve T cell anergy and has been found to be the most abundant chemokine present in ovarian cancer ascites (72). CCL17 and CCL22 skew T cells toward a Th2 direction (62, 63). In a study of ovarian cancers, Curiel and colleagues identified that TAMs and also tumor cells produce CCL22, which mediated the recruitment of T regulatory (Treg) cells to tumors (73). Treg cells were found to contribute to ovarian tumor growth by suppressing tumor-specific T cell immunity and to be predictive of poor patient survival (73). Reciprocally, Treg cells can promote TAMs as Treg cells express IL-4, IL-10, and IL-13 that induce M2 polarization of macrophages (74) (Figure 1).

Targeting of TAMs is still in its infancy, but has a strong application to ovarian cancer because macrophages are abundant in ascites. One potential strategy is to “re-educate” TAMs toward a tumoridical M1 phenotype. Inhibition of NF-κB signaling in TAMs has been found to induce an M2-to-M1 switch and lead to regression of ovarian tumor xenografts (75). Another possibility is to inhibit Stat3, which is activated in macrophages that are polarized toward an M2 phenotype by ovarian cancer ascites (68). Because of its ability to stimulate monocyte chemotaxis and M2 polarization, CCL2 is an attractive target. Treatment of mice bearing metastatic prostate cancer with CCL2 mAb has been reported to inhibit the overall tumor burden by 96% (76). Trabectedin is a DNA-damaging alkaloid that has been found to also inhibit CCL2 and IL-6 production and to inhibit differentiation of monocytes into macrophages (77). Selective toxicity of trabectedin for TAMs has been demonstrated in ovarian cancer xenograft models and in patient specimens (77, 78). Trabectedin in combination with pegylated liposomal doxorubicin (PLD) has been approved in Europe for treatment of platinum-sensitive recurrent ovarian cancer. In a pivotal Phase III trial (OVA-301), the combination of trabectedin and PLD was found to be more effective than PLD alone for patients with platinum-sensitive recurrent disease, with a higher response rate (35.3 vs. 22.6%) and increased PFS (median PFS 9.2 vs. 7.5 months) (79).

## Conclusion

The studies to date have revealed that the peritoneal cavity is a highly receptive environment for carcinomatosis, and that progression of ovarian cancer is dynamically orchestrated by a complex network of receptor/ligand-mediated interactions between tumor cells, resident peritoneal cells, and other host cells that are recruited to tumors. Several of these receptors and ligands are targeted by agents that are in clinical use, while others are under clinical development. Because many of the ligands stimulate multiple cell types, a priority for future studies is to delineate the impact on different cell populations of neutralizing these ligands. In addition, the effects of inhibitory agents on ovarian cancer cells need to be evaluated in solid tumor tissues and also in free-floating tumor cells. Furthermore, determining the optimal combinations of stromal-targeting agents with conventional chemotherapy or other targeted therapies and the appropriate clinical setting for their use are key priorities for future studies.

## Conflict of Interest Statement

The author declares that the research was conducted in the absence of any commercial or financial relationships that could be construed as a potential conflict of interest.

## Abbreviations

αSMA, α-smooth muscle actin; CAFs, cancer-associated fibroblasts; CCL, chemokine (C-C motif) ligand; CXCL, chemokine (C-X-C motif) ligand; ECM, extracellular matrix; EGF, epidermal growth factor; FABP4, fatty acid-binding protein 4; FAP, fibroblast activation protein; FGF, fibroblast growth factor; GFP, green fluorescent protein; HA, hyaluronic acid; IL, interleukin; LIF, leukemia inhibitory factor; mAb, monoclonal antibody; M-CSF, macrophage colony stimulating factor; MMP, matrix metalloproteinase; MSCs, mesenchymal stem cells; PDGF, platelet-derived growth factor; PDGFR, platelet-derived growth factor receptor; PLD, pegylated liposomal doxorubicin; TAMs, tumor-associated macrophages; TGF-β, transforming growth factor-β; TGFβRI, transforming growth factor-β type I receptor; TKI, tyrosine kinase inhibitor; Treg, T regulatory; VEGF, vascular endothelial growth factor; VEGFR, vascular endothelial growth factor receptor.
